# The Perspectives of Healthcare Professionals and Managers on Patient Involvement in Care Pathway Development: A Discourse Analysis

**DOI:** 10.1111/hex.14101

**Published:** 2024-06-10

**Authors:** Mildred Visser, Naomi ‘t Hart, Marleen de Mul, Anne Marie Weggelaar‐Jansen

**Affiliations:** ^1^ Erasmus School of Health Policy & Management Erasmus University Rotterdam The Netherlands; ^2^ Clinical Informatics Eindhoven University of Technology Eindhoven The Netherlands; ^3^ Tranzo, Tilburg School of Social and Behavioural Sciences Tilburg University Tilburg The Netherlands

**Keywords:** care pathway, co‐creation, co‐design, discourse analysis, patient involvement, qualitative research

## Abstract

**Background:**

The WHO advocates patient and public involvement as an ethical imperative, due to the value of the lived experience of patients. A deeper understanding of the shared meanings and underlying beliefs of healthcare professionals and managers for and against including patients in care pathway development.

**Objective:**

To explore the considerations of healthcare professionals and managers on the involvement of patients and public in care pathway development.

**Methods:**

In a medical rehabilitation centre we conducted a single case study that was part of a 2‐year action research programme on blended care pathway development. Following 14 semistructured interviews with healthcare professionals and managers, we analysed their discourses on the value of patient involvement as well as the potential threats and opportunities.

**Results:**

We identified four discourses. *Patient as expert* frames involvement as relevant, as adding new perspectives and as required to fully understand the patient's needs. *Skills and representation* is based on the construct that obtaining valuable insights from patients requires certain skills and competences. *Self‐protection* focusses on personal, interprofessional objections to patient involvement. *Professional knows best* reveals expertise‐related reasons for avoiding or postponing involvement.

**Conclusion:**

These discourses explain why patient and public involvement in care pathway development is sometimes postponed, limited in scope and level of participation, and/or avoided. The following strategies might minimise the paralysing effect of these discourses: strengthen the capabilities of all stakeholders involved; use a mix of complementary techniques to gain involvement in distinct phases of care pathway development; and create/facilitate a safe environment. Put together, these strategies would foster ongoing, reciprocal learning that could enhance patient involvement.

**Patient or Public Contribution:**

This study belonged to an action research programme on blended care pathway development (developing an integrated, coordinated patient care plan that combines remote, digital telehealth applications, self‐management tools and face‐to‐face care). Multidisciplinary teams took a quality collaborative approach to quality improvement (considering patients as stakeholders) to develop 11 blended care pathways. Although professionals and managers were instructed to invite patients onto their teams and to attend care pathway design workshops, few teams (3/11) actually did. Unravelling why this happened will help improve patient and public involvement in care pathway development.

**Abbreviations**
GDPR: General Data Protection Regulation
WHO: World Health Organization

## 1 Introduction

Patient and public involvement (PPI) in healthcare quality improvement is widely recognised as important and necessary, in both academia and daily practice as patients have the necessary expertise, given their lived experience of care services [1, 2, 3, 4]. The WHO advocates for the meaningful involvement of patients and informal caregivers through co‐creation, considering PPI an ethical imperative based on such principles as dignity, respect, equity, and inclusivity [2, 5, 6]. The literature reports greater acceptance of patients' views and experiences as valuable input to quality improvement, alongside the expertise of healthcare professionals [6, 7, 8, 9]. PPI is strategically relevant and a frequently stated goal of healthcare organisations [6, 10, 11]. However, it has proven challenging to create fruitful partnerships between healthcare professionals and patients [5, 9, 12, 13].

Although PPI comprises a broad research field, focussed mainly on shared decision‐making [14, 15, 16], quality improvement activities [11, 17, 18], and eHealth co‐design [19, 20, 21, 22], few studies focused particularly on PPI in care pathway (CP) development [23, 24, 25, 26, 27]. However, PPI in CP development offers a unique opportunity to reach a deeper understanding of the patient's experiences and needs as valuable input in quality improvement [9, 12, 23, 28]. PPI can be incorporated throughout CP development [5], such as the design, implementation and evaluation phases [7]. Even though proven challenging [28] by inviting patients to become engaged members of the multidisciplinary CP development team, a partnership between healthcare professionals and patients can be established [5, 7, 28, 29]. However, the literature points to the lack of a framework for effective strategies for patients' involvement in pathway development teams [6, 7, 9, 23]. Additionally, the well‐known CP development methods by Lodewijckx et al. [30], and Vanhaecht et al. [31]. do not address creating partnership between healthcare professionals and patients.

### 1.1 Research Objective and Aim

Taking a quality collaborative approach that considers patients stakeholders in the CP development process, 11 multidisciplinary teams involved in an action research programme developed blended CPs. Our study was inspired by observing that only 3 of the 11 teams, despite being part of the methodology, actually invited patients or their representatives to join the pathway design sessions. Given their crucial role in enabling PPI, and considering the scarcity of relevant literature, our aim was to explore the beliefs and considerations of healthcare professionals and managers working on CP development to obtain helpful insights that may improve PPI in this process.

### 1.2 Theoretical Background on Obstacles Patient Involvement

Several studies report a limited uptake of PPI in healthcare and criticise current efforts [5, 12, 13, 29]. According to Ocloo and Matthews [13] PPI is tokenistic when patients are not taken seriously. Various studies have evaluated three categories of obstacles to PPI in quality improvement. The first is project management issues, such as conflicting interests and uncertainty on how to incorporate the patients' input in decisions [15, 16, 17, 18, 19, 26, 27, 29, 32] lack of clarity on the role and expectations of patients [6, 7, 13, 19, 23, 26, 29, 33] and managerial issues (e.g., time and costs required [6, 11, 13, 16, 17, 18, 19, 27, 29, 32, 34, 35, 36] resource availability to support the process [13, 24, 26, 29, 34, 37, 38]). The second covers skills and knowledge as a preconditions for PPI, such as limited knowledge and co‐creating skills ([5, 6, 11, 13, 16, 18, 19, 23, 24, 26, 29, 33, 36, 37, 39, 40] and concerns about representativity [6, 13, 15, 17, 23, 27, 29, 40, 41, 42, 43]). The third category relates to social and personal issues, such as unequal relationships and power disparities [5, 6, 11, 13, 14, 18, 19, 25, 26, 27, 29, 32, 34, 35, 36, 44, 45]. Other social and personal issues concern the negative beliefs or attitudes of both professionals and patients towards PPI [5, 6, 13, 19, 24, 29, 33, 34], resistance of healthcare professionals to PPI [5, 13, 19, 29, 33, 34], patients' health status or health‐related constraints [13, 19, 29, 32, 33, 34, 36] and dealing with emotions [17, 29, 38]. In sum, the literature reports many PPI obstacles in quality improvement efforts, several of which relate to the opinions and belief systems of healthcare professionals and managers. However, the literature on PPI in CP development is limited [23, 24, 25, 26, 27].

## 2 Materials and Methods

This single‐case study was conducted as a sub‐study of a 2‐year action research programme on blended CP development in a Dutch medical rehabilitation centre (~800 employees, 16,000 patients and 16 locations) during which 11 multidisciplinary teams developed blended CPs. Two researchers, one of whom was not involved in the action research, collected data from members of the multidisciplinary teams. Each team set up a leadership triangle comprising a coordinator (rehabilitation practitioner, for example, physical therapist, speech therapist, social worker), medical leader (rehabilitation physician) and manager. Criterion sampling was used to gain insights from different perspectives [46]. Our sample included 11 leadership triangles containing four coordinators, five medical leaders and five managers (*N* = 14). Respondents were recruited by an e‐mail explaining the aim of the study and the data protocol. All respondents agreed to participate.

We employed discourse analysis for this qualitative study. Following Gee and Handford [47, p. 1], we define discourse analysis as ‘the study of language in use, the meanings we give language and the actions we carry out when we use language in specific contexts’. This approach allowed us to identify communication patterns and gain insight into how the used language shapes the views of PPI and subsequent actions of professionals and managers working on CP development.

One researcher, uninvolved in the action research programme (NtH), collected data in June 2022 and May 2023 from different pathway development respondents in audio‐recorded, semi‐structured interviews using a predefined topic guide (see Appendices S1 and S2). The interviews lasted between 15 and 45 min and took place either online via Microsoft Teams or in person on location. The interviews were transcribed verbatim and analysed thematically according to the methodology described by Braun and Clarke [48]. Two independent researchers (MV, NtH) familiarised themselves with the transcripts and then conducted an inductive coding process using ATLAS.ti 23. The open coding process was followed by grouping sets of related codes to reveal wider patterns (see Appendix S3). This process identified recurring language of interest, which led the whole research team to iteratively identify four discourses.

Ethical approval, including a check on GDPR compliance and Good Medical Practice compliance was obtained from the Erasmus University Ethical Review Committee (ETH2122‐0333). Informed consent was obtained before data collection. After the interviews were transcribed and pseudonymised, the transcripts were e‐mailed to the respondents who were given the opportunity to withdraw from the study.

## 3 Results

Our analysis identified four discourses that revealed the PPI belief systems and considerations of healthcare professionals and managers in the pathway design sessions: (1) patient as expert, (2) skills and representation, (3) self‐protection and (4) professional knows best.

### 3.1 Patient as Expert Discourse

The *patient as expert* discourse assumes that PPI is needed to understand what patients value due to their lived experience. It highlights their unique insights, which the professionals may lack. In this line of reasoning, excluding patients from the development process could result in a pathway less tailored to the patient's values.

*“*Patients have a completely different view on the matter. They're on the other side of the table.*”* Medical leader (C)

*“*It was very nice to get input from another perspective, because it creates different dynamics and, I think, results in a different care pathway.*”* Medical leader (M)

Respondents emphasise the importance of inviting patients to participate and of taking their opinions seriously. This can only be achieved when patients are granted an equal voice, as the next quote illustrates.

*“*If I were to involve [patients], it would be as equals. […] Perhaps sometimes with even a more decisive say than therapists because they've experienced it themselves. I'd give them a decent say.*”* Manager (P)

This discourse regarded the extent of patient influence as relevant, emphasising that although their voices should be taken seriously not all of their suggestions should be automatically adopted. Respondents pointed out that in the team's decision‐making process, the patient's opinion is as important as the professionals opinion. Respondents also mentioned the importance of balancing the opinions because they were aware of organisational constraints, such as finance and capacity, that could prevent the realisation of patients' preferences.

*“*The individual patient's perception is really important, for sure. But that doesn't mean that we can fulfil their every wish. There is a bigger picture. It must be financially viable and achievable in terms of capacity.*”* Coordinator (K)

Respondents pointed out that inviting patients to join the CP development team could raise the false expectation that, for example, their opinion would be or should be favoured. Thus, it requires a delicate balance between managing patient expectations of the outcome of their involvement and taking their input seriously.

*“*We practitioners have to deal with those limitations… and, well, patients have to deal with them too. If they say, ‘We think [the duration of a hospital stay] is too short, we'd like it to be three times longer’, then—sorry—that's simply impossible. There are boundaries.*”* Medical leader (B)

### 3.2 Skills and Representation Discourse

The *skills and representation* discourse is based on the construct that if patients are going to make a valuable contribution to CP development, they will need certain skills and competences. This line of reasoning demands carefully selecting which patients will be invited to join the multidisciplinary team, thus limiting involvement to certain qualified patients.

*“*I don't think everyone is suited to participate in such a project. You have to have some, yes what is it, qualities, background, communication skills, and so on.*”* Coordinator (D)

Respondents felt that there should not be any language barriers to full participation on the multidisciplinary team, that is patients should be able to speak Dutch, and they should be able to abstract their own experience. Several respondents linked the required competences and ability to abstract to a threshold at the educational level.

*“*When you start working together on a project, of course it's helpful if someone can look beyond their own problems and not just talk from their own viewpoint, but say in this case, from the perspective of anyone with a spinal cord injury.*”* Coordinator (D)

*“*…to put it very bluntly, [inviting] the higher educated, native‐Dutch‐speaking patients who can provide a bit more information and can also think more on both the abstract and general levels.*”* Medical leader (H)

In medical rehabilitation settings, healthcare professionals work with vulnerable patients. When respondents were considering whether a particular patient could be involved in developing the CP, they were concerned with the degree of strain and stress that the patient could handle. Respondents tentatively expressed their concern about patients with conditions such as ALS (Amyotrophic lateral sclerosis) or MS (Multiple Sclerosis), or those affected cognitively and weighed the benefits of PPI against the risk of overdemanding the patient.

*“*Our target group has cerebral disorders and they have to be able cope with [PPI]. So I think that would be an obstacle because you have to assess which patient can handle [being involved].*”* Coordinator (A)

Reflecting on the required competences and educational level of patients, respondents know how carefully they're selecting the ‘right’ patients and how this creates a bias. They spontaneously raised the question of representativity, as the target population of a CP is more heterogeneous:

*“*On the one hand, you prefer people who are capable of thinking along, taking part in a group setting and providing input. On the other hand, they have to represent their entire target group.*”* Medical leader (M)

*“*If we only invite the highly educated patients… well, that's a very specific selection.*”* Medical leader (M)

### 3.3 Self‐Protection Discourse

The *self‐protection* discourse focusses on personal, interprofessional objections to PPI in CP development. Respondents said that they avoided or postponed involving patients because they (or their colleagues) fear criticism, feel vulnerable and feel pushed outside their comfort zone, which might lead them to be less than frank and forthright in discussions. Patient participation in CP development demands a certain personal receptivity, trust, and a willingness to show vulnerability from every healthcare professional involved.

*“*I think it can be scary at times because you're putting yourself in a vulnerable position. You're giving the patient room to express their opinion about how things could be done differently. Often, it's a case […] of focusing on negative things, whereas it's nice to pay attention to the patient's positive experiences too. […] Well, that [negative comment] feels like an attack and not everyone fancies that. So that's a barrier.*”* Medical leader (C)

Respondents who were open to PPI stated that having a patient present is undesirable whenever healthcare professionals have a difference of opinion, especially in the developmental phase when, for example, variations in current care approaches need to be harmonised and emotional outbursts can be expected.

*“*Well, in this stage, when healthcare professionals are almost at each other's throats, it's not useful to involve a patient. […] The professionals would feel inhibited and it's certainly not easy to get everyone to agree. […] So yes, this is not a good time to involve patients.*”* Medical leader (B)

### 3.4 Professional Knows Best Discourse

The *professional knows best* discourse focusses on avoiding or postponing PPI in CP development because, in the professional's opinion they do not need the patient's ‘expertise’ (on their own experience). Some respondents felt that some professionals have a know‐it‐all attitude, thinking they know best what patients need in the CP.

*“*Well, the biggest threshold is […] the healthcare professionals' ego. Thinking that you know it all so well, so why should you change anything? That's the biggest pitfall.*”* Medical leader (C)

As this quote shows, because of their routine contact with patients and the feedback they get from patients during treatment sessions, healthcare professionals feel they already know and understand the patient perspective. This point of view questions the added value of PPI in CP development.

*“*As a team, we think we're doing quite well. Not because it's our own point of view, but because of the feedback from patients.*”* Medical leader (D)

In addition, some respondents feel that patients are not in a position to contribute when a multidisciplinary team is working on implementing evidence‐based medicine/nursing or solving financial issues. These respondents implicitly link the added value of PPI to service and communication‐related topics, rather than to the content or logistics of the patient's care.

*“*I find this hard, because when I look back on the process of developing our care pathway modules, I don't see much added value in [PPI]. I imagine that patients would question their added value if the conversation is [only] about certain treatment plans and treatment frequencies. I wouldn't have known when to involve them.*” Coordinator* (A)

In this view, it is not necessary to involve patients in the design phase of CP development. The best moment to involve them is when the pathway is ready.

*“*Once you're in the implementation and evaluation phase, [PPI] seems fine to me. [Then] in several locations you select patients with a broad perspective and ask them things like: ‘What do you think of this or that?’, ‘How do you rate the choices we made?’ and ‘What do you think we could do differently?’. Yeah, so after the implementation phase and actually in the evaluation phase.*”* Medical leader (B)

Consequently, when patients are invited to participate later on in the development process, their involvement is framed differently. For example, they are regarded more as advisers or consultants than as partners in the CP development process.

*“*Well, we're not going to invite patients all the time, but we are going to ask them now and then how they feel about this [the proposed care pathway] and what they think of it.*”* Manager (R)

## 4 Discussion

### 4.1 Comparison With Literature

This study provides a deeper understanding of healthcare professionals' and managers' discourses on PPI in developing CPs. We identified four discourses (Table 1) on patient involvement and showed how these views affected PPI. The four discourses align with the literature on PPI obstacles PPI in several ways.

**Table 1:** Four discourses on patient involvement in care pathway development.

Discourse	1) Patient as expert	2) Skills and representation	3) Self‐protection	4) Professional knows best
Position of professional/manager on PPI partnership	PPI is essential to take patients' values into account.	PPI has limited value as patients lack skills or do not represent the group.	PPI puts the professional in a vulnerable position.	PPI is not an addition to professionals' expertise.
Aspects of the belief system	Patients bring unique insights. Patients and professionals contribute equally to decision‐making. Taking patients seriously is not the same as granting all their wishes.	To be valuable, PPI requires skills, a broad outlook, certain education and health status. Patient selection criteria result in debatable representativity.	Professionals must discuss some sensitive topics among themselves first. PPI means vulnerability and receiving criticism which disturbs the relationship between patient and professional.	Professionals know best what is needed. PPI is only suited to specific components of the pathway. PPI is only useful after the design phase of CP development.
Resulting PPI action/inaction	PPI is initiated naturally with high level of participation, and clear boundaries.	PPI is avoided, postponed, or limited in level of participation and/or scope.	PPI is avoided or postponed.	PPI is avoided, postponed, or limited in level of participation and/or scope.

First, the *skills and representation* discourse turns PPI into a problem because, on the one hand, according to the respondents, patients involved in CP development are supposed to need certain skills/competences to contribute to a fruitful, equal discussion. On the other hand, in their opinion, patients should represent the ‘average’ patient (who often lacks these skills) to provide more than anecdotal input. The respondents find these two requirements contradictory, as they exclude people lacking certain skills, and suggest that PPI has limited value when they are included. This aligns with other research on PPI barriers [6, 13, 23, 29]. While patient representation and representativeness are entangled in the discourse of professionals and managers in our study, it is important to distinguish patient representation in the project team from representativity of the target population in CP development [49], as each has its own strategies to overcome PPI barriers.

Representation in the CP development team of all stakeholders involved in the care process (including patients) is paramount from a participatory project management perspective to ensure the availability of a variety of perspectives [28, 50]. Although common in participatory design methods (such as human‐centred and experience based design), in the CP development methods this isn't common yet [30, 31, 50, 51]. Regarding representation, the respondents argue in this discourse that patients need certain skills and competences when participating in a CP development team. Cox et al. [5] support the idea that a successful PPI partnership requires capable knowledge, skills and attitudes in all stakeholders, including patients. However, it is a misconception to place the responsibility of representativeness on the patient representative in the CP development team because, paradoxically, using advanced selection criteria immediately reduces representativity and complicates the recruitment and selection of potential participants. Scholars such as Maguire et al. [40] and Scholz et al. [41, 42] argue that the requirement based on representativeness may even have the opposite effect, leading to *dis*empowerment of patient representatives and *de*legitimisation of their involvement. However, to develop a CP tailored to the target population's needs and values the diverse input of a variety of patients benefits the developmental process. This need for CP representativity can also be addressed with a combination of complementary techniques to gain PPI in all phases of the CP development process: focus groups, shadowing, interviews, using patient‐reported experience data, and surveys [9, 28, 52]. This ‘triangulation’ of patient perspectives will reduce the unrealistic expectation of professionals and managers in the skills and representation discourse that the patient involved in CP development has to be ‘the’ patient representative.

Second, our study addresses the issue of unequal relationships and power dynamics [6, 14, 25, 35, 53]. Discourses 2–4 are built, albeit implicitly, on the premise of a desirable distance between the healthcare professional and patient. In the *professional knows best* discourse, respondents assume that they know what patients need, based on their daily patient encounters and professional expertise. However, their interpretation of patient/public needs may be subject to personal bias [6, 14, 25, 35, 53]. According to Ocloo and Matthews [13], critical selection of participants, as the *skills and representation* discourse reveals, is in itself a manifestation of power imbalance. Additionally, respondents argue in the *self‐protection* discourse that healthcare professionals may need to discuss sensitive topics amongst themselves first. They may need to shield patients from differences of opinion or shield their own vulnerability as professionals susceptible to criticism from patients or public. This also points to a power imbalance. In contrast, some scholars argue that involving patients in CP development is a way to address the power dynamics. Ocloo et al. [35] argue that although ‘power dynamics act as contextual constraints limiting patient experience improvements at every level of the system’ [35, p. 11]. It is a capability that requires development or the application of specific methodological approaches such as experience‐based co‐design and co‐production.

Third, we see that discourses 2–4 reflect timing and participation level issues justifying postponement, restricted scope or participation and even rejection of PPI. Respondents mentioned the importance of excluding patients whenever professionals needed the time to align or resolve conflicts. Implicitly, these discourses reveal the assumption that PPI is less useful in the design phase and more useful in the experimentation, implementation and evaluation phases, a notion that links to the obstacles concerning the patients role described in literature [6, 7, 13, 19, 23, 26, 29, 33]. Consequently, the level of PPI lowers from partnership or collaboration to the level of consultation, as outlined in the framework for PPI by Carman et al. [33] and the IAP2 public participation spectrum [54, 55]. Furthermore, lowering the participation level and shifting PPI to begin after the design phase risks limiting PPI to ‘safe’ topics (not disputed professionally), such as service aspects, patient information and communication strategies, or simply to tokenism [5, 9, 12, 13]. While these are real risks, various strategies exist to engage patients and the public at all stages, so this situation can be improved. We elaborate on this in the implications for practice.

### 4.2 Patient Involvement Rationale

The utilitarian rationale for PPI views the use of patients' experience as a tool to improve the value and effectiveness of healthcare services [2, 56, 57]. This is in line with the reasoning of the respondents in the *patient as expert* discourse that frames PPI as benefiting from the valuable input of the patient's lived experience and fresh perspective. The patients experiential knowledge complements expert knowledge [2, 7, 13, 16, 23, 56, 58] resulting in a pathway crafted onto what patients value. It is widely acknowledged that patients have a crucial role in healthcare improvement, based on their personal lived experience [1, 2, 3, 4].

There is, however, another justification for PPI, which dominates policy documents: the empowerment rationale. This states that patients have a rightful role ‘to be involved in decisions that affect them and their lives […] and that this process will empower them’ [56, p. 124]. The empowerment rationale emphasises the importance of equity and patient empowerment [2, 35, 56]. Moreover, it frames meaningful PPI as an ‘ethical imperative’ that embraces the principles of respect, equality and inclusivity [2, 5, 6]. Strikingly, empowerment played a minor role in the *patient as expert* discourse. Both the literature and the WHO emphasise adherence to the empowerment rationale as the foundation of equal contribution by patients and the basis of co‐creation in partnership. This implies that patients are important stakeholders in all CP development phases: from the initial design phase (understanding what change is needed), through the phases of experimentation and implementation in mundane practices, up to evaluation and readjustment. However, according to Farr [25] establishing an equal partnership is not guaranteed as it requires constant critical reflection and dialogue on the dominating practices and empowering processes that occur in the CP development team. We hope the insights derived from the four discourses can contribute to this critical reflection and foster a dialogue on the assumptions to (not) include patients in the entire CP development process. As the utilitarian rationale for PPI appears to be dominant in our study, the results align with Draper and Rifkin's [56] findings on person‐centred health systems. One explanation for this dominance might be that research and policy documents describe PPI from a more ideological point of view, whereas our respondents expressed a more pragmatic view. Morgan [57] has also described this difference between the utilitarian pragmatists and the empowerment activists. Nevertheless, according to the WHO [2], involvement initiated by those in power (induced participation), such as PPI in CP development, can empower and support patient autonomy as induced participation can strengthen the capabilities [5] of both individuals and communities. Further research could address the objective of gaining a better understanding of the difference in rationales for PPI between healthcare professionals, research and policy documents, between the pragmatists who favour utilitarian models and the activists who prefer empowerment models [57].

### 4.3 Implications for Practice

Our analysis of the discourses shed valuable light on why PPI in all phases of CP development is not easy to accomplish, as other scholars have also outlined [5, 12, 13]. To establish valuable PPI, healthcare organisations should consider the impact of the discourses on PPI practice when multidisciplinary teams are developing CPs. The negative effects described in discourses 2–4 can be minimised with the following research‐based strategies.

Successful partnerships regard PPI as a mutual and continuous learning process based on the principles of equality and power sharing [5]. We agree with Cox et al. [5] that this requires stakeholders to also have capability: the ability to use these competences in unpredictable situations and in interaction with different stakeholders. The learning process can develop the necessary competences and simultaneously grow into shared power [5, 35], but it requires time, and the patience and dedication of all stakeholders. Not only the patient needs to learn, but the professional as well. This emphasises the need for organisations to implement various approaches to stimulate the learning process [5, 59]. Bombard et al. [6] mention such strategies as training sessions to clarify roles and develop skills. Also, when viewed like this, the selection criteria mentioned in the *skills and representation* discourse are turned into learning goals. Representativity is enhanced by the participation of patients from different backgrounds. As discussed above, representativity can also be addressed with a combination of complementary techniques to gain PPI in all phases of the CP development process: focus groups, shadowing, interviews, using patient‐reported experience data, and surveys [9, 28, 56].

Developing a framework for PPI in all phases in CP development would be beneficial in supporting organisations to involve patients in all CP development phases, from partnership to consultation level. This framework could build on existing CP development methods [30, 31], research on participatory methodologies in co‐creation of technology [60], or in research [61]. However, it is important to consider the applicability, implementability, and practicality of this framework, since CP development processes are complex and time‐consuming. Each organisation has its own unique context, constraints and challenges. Moreover, the PPI discourses demonstrate individual (and collective) belief systems, which in principle are not easy to change. Both the *self‐protection* and the *professional knows best* discourses avoid PPI. However, instead of avoiding involving patients, more emphasis on creating a safe environment and equality is needed for multidisciplinary teams to discuss every CP development topic in the presence of patients.

In their capability framework for successful partnerships in quality improvement Cox et al. [5] address the domain ‘relationships and communication’, which includes the capabilities ‘working and learning as a team with strong conflict resolution’ and ‘collaborating and communicating’ [5]. These are especially at stake when professionals must find consensus on topics that lead to differences of opinion. Although we agree with Chambers [62, p. 18] that ‘conflict can be seen as an essential and creative factor in change for the better’, and it is best to anticipate conflict to be productive in PPI activities [57], such a safe environment should be created and facilitated through dedicated activities. Bombard et al. [6] offer strategies to facilitate this, such as democratic dialogues, exercises on values and beliefs, and training sessions to increase sensitivity and reduce power imbalance. Again, it is clear that project teams should allocate sufficient time to develop trusting relationships.

### 4.4 Study Strengths and Limitations

A strength of our study is that the researcher collecting the data was unknown in the organisation and uninvolved in the CP development project. This ensured a safe space for respondents to give their honest opinions and prevented the researcher bias associated with ‘going native’ in action research [63]. To enhance credibility, two independent researchers strived for investigator triangulation [64, 65] by coding the data in an iterative process involving reading and re‐reading the transcripts.

A few limitations need to be addressed. The transferability of our findings depends on context similarity [46]. As this was a single‐case study in one rehabilitation centre, we recommend repeating our study in other healthcare organisations not just to confirm our findings but also to collect data at various points to study change over time in a continued discourse analysis. The strategy chosen for criterion sampling might have led to selection bias. However, we minimised this effect by including both stakeholders who have experienced PPI in their CP development process and those who have not. Also, saturation might not have been reached because the criterion sample was maximised by the available number of leadership triangles and participation was voluntary. Hence, we suggest further research on PPI discourses. Because we focussed on the perspective of professionals and managers, it would be interesting if future research contrasted the professional/manager perspective with the patient/public perspective in the same CP development initiative.

## 5 Conclusion

The four discourses we identified (Table 1) showing the belief systems and considerations of healthcare professionals and managers on PPI in the pathway design sessions shed valuable light on why PPI gets postponed, restricted or avoided. As respondents may adopt more than one discourse, this gives us a multilayered perspective that allows us to conclude that establishing a PPI partnership may be regarded as a continuous, mutual learning process. The selection criteria and concerns about patient representation may be addressed in all CP development phases to meet the challenge of enhancing equality by moving PPI beyond the consultation level only. Another challenge relates to the vulnerability of professionals and their perceived power imbalance in this process. We suggest strategies that may help practitioners and managers overcome these challenges: provide training to strengthen stakeholders' capabilities and encourage prolonged interaction; combine complementary techniques to increase PPI in different phases of the CP development phases; create a safe environment where differing opinions and even conflict can be anticipated and used effectively. Taken together, these strategies should foster the ongoing, reciprocal learning that will help realise the goal of healthcare organisations to use valuable PPI input for health service quality improvement.

## Author Contributions

**Mildred Visser**: conceptualisation, writing–original draft, writing–review and editing, methodology, formal analysis, project administration, data curation. **Naomi ‘t Hart**: investigation, writing–original draft, formal analysis. **Marleen de Mul**: writing–review and editing, supervision, writing–original draft. **Anne Marie Weggelaar‐Jansen**: conceptualisation, methodology, supervision, funding acquisition, writing–original draft, writing–review and editing.

## Conflicts of Interest

The authors declare no conflicts of interest.

## Supporting information

Appendix A: Topic list EN.

Appendix B: Topic list NL.

Appendix C: Code groups and codes NL and EN.

## Data Availability

The data that support the findings of this study are available on request from the corresponding author. The data are not publicly available due to privacy or ethical restrictions.
