# Supplementary material for: The Perspectives of Healthcare Professionals and Managers on Patient Involvement in Care Pathway Development: A Discourse Analysis
Source: Health Expect. 2024 Jun 10;27(3):e14101. doi: 10.1111/hex.14101 (PMC11163266; doi:10.1111/hex.14101)
Supplement: Supplementary file 1 — Appendix A: Topic list EN. [file HEX-27-e14101-s003.docx]

**Appendix A: Topic list**

|  | **Topics** | **Subtopics** |
| --- | --- | --- |
| What | Care pathway | Patient group  Goal and objective  Process  Evaluation |
|  | Patient participation | Definition |
| Why | Value of patient involvement | Experiential knowledge  Improving quality  Legitimacy  Normative argument  Patient-centered |
| How | Ways of involving patients | Focus groups/interviews/Surveys/Participation in meetings  One time/Multiple times/Continuous involvement  Patients/Patient representative  Development/Implementation/Evaluation/Follow-up |
|  | Level of involvement | Consultation/Involvement/Partnership  Patient-characteristics  Care pathway characteristics  Shared power/Power is with the patient/Power is with the professionals |
|  | Barriers | Power imbalance  Knowledge imbalance  Lack of clarity on expectations  Lack of information  Attitude of professionals  Recourse and time constraints  Inadequate design strategy |
|  | Overcoming barriers | Training  Informing  Expectations management  Stimulating reflexivity |
