# Supplementary material for: The Perspectives of Healthcare Professionals and Managers on Patient Involvement in Care Pathway Development: A Discourse Analysis
Source: Health Expect. 2024 Jun 10;27(3):e14101. doi: 10.1111/hex.14101 (PMC11163266; doi:10.1111/hex.14101)
Supplement: Supplementary file 2 — Appendix B: Topic list NL. [file HEX-27-e14101-s002.docx]

**Appendix B: Topic list**

|  | **Topics** | **Sub topics** |
| --- | --- | --- |
| Wat | Zorgpad | Patiëntengroep / doelgroep  Doel  Proces  Evaluatie |
|  | Patiënt participatie | Definitie |
| Waarom | Waarde van patiënt participatie | Ervaringsgerichte kennis  Kwaliteit verbeteren  Legitimiteit  Normatief argument  Patiëntgericht |
| Hoe | Manieren om patiënten te betrekken | Focus groepen/interviews/vragenlijsten/Participatie in bijeenkomsten  eenmalig / meerdere keren /Continue  Patiënten /Patiëntvertegenwoordigers  Ontwikkeling/Implementatie/Evaluatie/Follow-up |
|  | Niveau van participatie | Consultatie/Betrekken/Partnerschap  Patiënteigenschappen  Zorgpad eigenschappen  Gedeelde macht / Macht ligt bij de patiënt / Power ligt bij de professionals |
|  | Barrières | Macht ongelijkheid  Kennis ongelijkheid  Onduidelijkheid over verwachtingen  Gebrek aan informatie  Houding van professionals  Beperkingen in capaciteit en tijd  Inadequate ontwerp strategie |
|  | Barrières overwinnen | Training  Informeren  Verwachtingsmanagement  Stimuleren van reflexiviteit |
