# Supplementary material for: The Perspectives of Healthcare Professionals and Managers on Patient Involvement in Care Pathway Development: A Discourse Analysis
Source: Health Expect. 2024 Jun 10;27(3):e14101. doi: 10.1111/hex.14101 (PMC11163266; doi:10.1111/hex.14101)
Supplement: Supplementary file 3 — Appendix C: Code groups and codes NL and EN. [file HEX-27-e14101-s001.pdf]

Appendix C - Code groups & codes

Document Group	Codes NL	Codes EN
D1: Patient as expert	1. Ander perspectief<br>1. De wil is er<br>2. Gelijkwaardige stem<br>3. Ruimte geven - niet in beton<br>4. Verwachtingsmanagement & kaders	1. Alternative perspective<br>1. Willingness/Commitment<br>2. Equal voice<br>3. Give space – not set in stone<br>4. Expectations management & limitations
D2: Skills & Representation	1. Profiel - Behandelrelatie<br>1. Profiel - Brede blik – overstijgend<br>1. Profiel - Kritisch durven zijn, iets brengen<br>1. Profiel - Langer uit revalidatie<br>1. Profiel - Patiëntvereniging<br>1. Profiel - Positief profiel<br>1. Profiel - Relatie hebben<br>1. Profiel - Vaardigheden en opleidingsniveau<br>1. Profiel - Voorbij 1 ervaring<br>1. Profiel - Zit niet in systeem<br>1. Profiel - Zorg kennen	1. Profile - Treatment relationship<br>1. Profile – Broad, transcending perspective<br>1. Profile – Being critical, to contribute something<br>1. Profile – Rehabilitation in the past<br>1. Profile - Patient association<br>1. Profile - Positive profile<br>1. Profile - Having a relationship<br>1. Profile - Skills and level of education<br>1. Profile - Beyond a single experience<br>1. Profile - Not in the system<br>1. Profile - Familiar with health system
	2. Belasting patiënt<br>2. Cognitie	2. Patient burden<br>2. Cognition
	3. Is één mening<br>3. Representativiteit<br>3. Selectief kiezen patiënten	3. Having only one opinion<br>3. Representativeness<br>3. Selective choice of patients
D3: Self-protection	1. Gêne - onderling gedoe<br>2. Angst voor kritiek<br>3. Kwetsbaarheid door transparantie<br>4. Uit je comfortzone<br>5. Regie loslaten	1. Discomfort - interpersonal issues<br>2. Fear of criticism<br>3. Being transparent results in vulnerability<br>4. Stepping out of your comfort zone<br>5. Let go of control
D4: Professional knows best	1. Meerwaarde twijfel<br>1. Zelf beter weten - we doen het goed<br>2. Onderwerp niet geschikt<br>3. Expertise - zelf bespreken	1. Doubting the added value<br>1. Knowing better oneself - we're doing right<br>2. Subject not suitable<br>3. Expertise – discussing it ourselves
